# Validation of the Chinese version of academic goals orientation questionnaire in nursing student: a study based on SEM and IRT multidimensional models

**DOI:** 10.1186/s12912-023-01630-0

**Published:** 2023-12-06

**Authors:** Yuqing Li, Lei-lei Guo, Jiaofeng Gui, Xiaoyun Zhang, Ying Wang, Haiyang Liu, Jinlong Li, Yunxiao Lei, Xiaoping Li, Lu Sun, Liu Yang, Ting Yuan, Congzhi Wang, Dongmei Zhang, Huanhuan Wei, Jing Li, Mingming Liu, Ying Hua, Lin Zhang

**Affiliations:** 1 Department of Graduate School, Wannan Medical College, 22 Wenchang West Road, Higher Education Park, Wuhu City, An Hui Province P.R. China; 2 Department of Surgical Nursing, School of Nursing, Jinzhou Medical University, No.40, Section 3, Songpo Road, Linghe District, Jinzhou City, Liaoning Province P.R. China; 3 Student health center, Wannan Medical College, 22 Wenchang West Road, Higher Education Park, Wuhu City, An Hui Province P.R. China; 4 Department of Occupational and Environmental Health, Key Laboratory of Occupational Health and Safety for Coal Industry in Hebei Province, School of Public Health, North China University of Science and Technology, Tangshan, Hebei Province P.R. China; 5 Obstetrics and Gynecology Nursing, School of Nursing, Wannan Medical College, 22 Wenchang West Road, Higher Education Park, Wuhu City, An Hui Province P.R. China; 6 Department of Emergency and Critical Care Nursing, School of Nursing, Wannan Medical College, 22 Wenchang West Road, Higher Education Park, Wuhu City, An Hui Province P.R. China; 7 Department of Internal Medicine Nursing, School of Nursing, Wannan Medical College, 22 Wenchang West Road, Higher Education Park, Wuhu City, An Hui Province P.R. China; 8 Department of Pediatric Nursing, School of Nursing, Wannan Medical College, 22 Wenchang West Road, Higher Education Park, Wuhu City, An Hui Province P.R. China; 9 Department of Surgical Nursing, School of Nursing, Wannan Medical College, 22 Wenchang West Road, Higher Education Park, Wuhu City, An Hui Province P.R. China; 10 Rehabilitation Nursing, School of Nursing, Wannan Medical college, 22 Wenchang West Road, Higher Education Park, Wuhu City, An Hui Province P.R. China

**Keywords:** Academic goals orientation questionnaire, Nursing, Students, Reliability, Validity, Structural equation modeling, Item response theory

## Abstract

**Objective:**

To translate the Academic Goals Orientation Questionnaire (AGOQ) into Chinese and to determine the validity and reliability of the (AGOQ) in Chinese nursing students based on SEM and IRT multidimensional models.

**Methods:**

The participants were 654 nursing students with an age range of 17–26 years (mean age 21.61 ± 1.73 years). The psychometric properties of AGOQ were investigated based on a dual analytical perspective of structural equation modeling (SEM) and item response theory (IRT).

**Results:**

The Cronbach’s α value of the questionnaire is 0.895. A four-factor model was obtained by exploratory factor analysis, which explained the variance of 71.892%. With confirmatory factor analysis, a new four-factors model was built and showed an acceptable goodness-of-fit, chi-square/degree of freedom (CMIN/DF) = 4.008, goodness of fit index (GFI) = 0.932, adjusted goodness of fit index (AGFI) = 0.905, comparative fit index (CFI) = 0.952, incremental fit index (IFI) = 0.952, Tucker Lewis index (TLI) = 0.941. In the analysis part of IRT, according to the comparison between Akek’s information criterion (AIC) and Bayesian information criterion (BIC), we choose the Graded Response Model (GRM) for analysis. The results show that the difficulty value is monotonically increasing, and the discrimination of all items is greater than 0.19, which shows that 16 items can be retained.

**Conclusions:**

This study tested the psychometric characteristics of AGOQ of nursing students in China. The results confirmed that the Chinese version of AGOQ has good psychometric characteristics and can be used to measure the academic goal orientation of nursing students in China.

**Supplementary Information:**

The online version contains supplementary material available at 10.1186/s12912-023-01630-0.

## Introduction

The national standard for teaching quality of undergraduate majors in colleges and universities [1] required that nursing students should have the basic ability of independent learning and innovative development, and be able to adapt to the changing social health care needs. At the same time, it is necessary to mobilize teachers’ subjective initiative, improve students’ active learning, and actively carry out student-centered teaching aimed at improving students’ autonomous learning ability and innovation ability. In China, the role of learning goal orientation has also been brought into play in teaching in various fields. For example, when setting course objectives, the learning objectives of nursing specialty will be divided into three aspects: knowledge objectives, skills objectives and attitude objectives. Through the evaluation of the effect of achieving the goal, teachers can know the students’ mastery and curriculum preference in time and give professional guidance to the greatest extent.

Academic goals were defined as the content and direction of one’s motivation for academic success or failure [2, 3], which were divided into four types of goals [4]: (i)learning or task goals, (ii)ego self-enhancement goals, (iii)ego self-frustration goals, and (iv)work avoidance goals. Research on different types of academic goals has traditionally considered learning and performance [5]. Goal orientation was based on achievement motivation goal theory. The goal perspective theory of achievement motivation [4] focused on identifying different types of goal orientations among students. The view that there were two goals had received special attention. These viewpoints were called **task-oriented** and **self-oriented** [6, 7]. However, some researchers also suggested that students may be avoidance-oriented in learning situations. Factor analysis showed that task orientation, self orientation and avoidance orientation are different goal orientation factors. In 1997, Norwegian scholars [4] studied a prediction among Norwegian students in grades 6 and 8, that was, self-orientation had different dimensions (self-frustration and self-enhancement), which may be separated from other goal orientations. There was a weak correlation between self-frustration and self-enhancement, and both dimensions were independent of task orientation. And they were related to academic achievement. In addition, Nicholls et.al [7] suggested that, as mentioned above, students may be evasive in learning situations. The measurement of job avoidance showed high reliability [7], and factor analysis also showed that it can be separated from task orientation and self-reinforcement orientation. The above results were verified by students in 2012 [8] and 2020 [9], and the final academic goal orientation was determined as four dimensions, namely, ego self-frustration goal, ego self-enhancement goal, work avoidance goal and learning or task goals.

First, students with Type I goals (learning or task goals) focused on intrinsic stimuli and sought to absorb knowledge, acquired skills, and gained a true understanding of the problem [10]. In short, they wanted to learn and improve their skills, so they were also called task-focused goals. Second, students with Type II and III goals (ego self-enhancement goals, ego self-frustration goals) were social in nature and students tried to satisfy external needs through academic achievement. Ego self-enhancement referred to seeking favorable outcomes, and ego self-frustration referred to having a defensive attitude and seeking to avoid setbacks and unfavorable images [4]. Both types of academic orientation had a social component. In other words, students sought social, academic, or family approval either to be better than their peers or to conceal mediocre performance, rather than to satisfy their intellectual needs [11]. Finally, the Type IV goals (work avoidance goals) referred to students avoiding learning activity engagement by using customary avoidance behaviors, such as expending minimal effort and avoiding complex tasks [12].

Barkur et al. [13] examined the correlation between learning goal orientation and academic performance and concluded that students with lower grades tended to engage in work avoidance compared to students with higher grades. The result was similar to those obtained by Palos et al. [14] among nursing students.

However, no Chinese studies on this topic were found during the literature search, possibly due to a lack of validated tools to measure students’ orientation toward academic goals. Academic goals orientation questionnaire (AGOQ) was first developed by a Norwegian scholar [4], and was translated into Spanish in 2012 [8] and applied to nursing students for the first time. In 2020, Manrique-Abril FG et al. [9] conducted a second verification on nursing students in Colombia (the official language is Spanish). The results showed that the questionnaire has sufficient validity and reliability in the Colombian context and can be applied to nursing students. In addition, the research on the academic goals of nursing students was helpful helpful in determining their academic orientation, thus becoming an auxiliary tool for teachers to select students and adjust the course content accordingly.

Therefore, this study aims to translate the Spanish version of the academic goals orientation questionnaire (AGOQ) into Chinese and evaluate the psychometric properties of the AGOQ in Chinese nursing students based on SEM and IRT multidimensional models.

## Methods

### Design and sample

Cross-sectional design and multi-stage sampling design were adopted in this study. From March to June 2023, a survey was conducted among nursing students in medical schools in Jinzhou, Liaoning Province, China. The investigators of this study are mainly nursing graduate students who conducted this study. They received unified training on how to use standardized language and guidance (Supplementary material 1 is the training guidance of investigators). All participants completed the test voluntarily. Inclusion criteria: (1) Full-time nursing students in school; (2) informed permission and voluntary involvement in this study; (3) Students who understand and voluntarily join this study. Exclusion criteria: (1) Students who are dropping out of school; (2) Students who are unwilling to participate in this study [15, 16].

According to Kendall’s working principle [17], the sample size is calculated using a rough estimation method of 10–20 times the number of variables. The survey questionnaire for this study includes 4 general demographic data items, 16 items of academic goals orientation questionnaire. A total of 20 variables needs to be analyzed. Considering the possibility of loss or invalidity during the sample recycling process, the sample size should be expanded by 20%, and the final sample size should not be less than 480 people. Finally, we collected 654 valid questionnaires.

### The instrument

The AGOQ has 16 items and four factors that pose questions to guide student learning. Items were divided into four dimensions based on the type of academic goal orientation, namely (i) Ego self-frustration goal (items 4, 7, 11, 14), (ii) Ego self-enhancement goal (items 2, 6, 10, 3), (iii) Work avoidance goal (items 3, 8, 12, 15), and (iv) learning or task goals (items 1, 5, 9, 16). A five-point Likert scale was used to mark the answers that best matched the subjects’ current state (1 = strongly disagree, 5 = strongly agree). The reliability of the original scale with Cronbach’s alpha (α) > 0.8 in all dimensions was adequate [4]. The total content validity index was 0.72 and had sufficient internal consistency [8].

### Translation procedure

There were various phases in the translation guide [18–20]. First, two multilingual expert translators translated the AGOQ from Spanish to Chinese. The Chinese version was translated into English by two more multilingual expert translators. Second, a multilingual panel of four nursing professionals and two psychologists examined each item’s cultural and language equivalency. A preliminary test was given to 30 nursing students. The AGOQ was changed based on their comments. Supplementary material 2 shows the item of AGOQ.

### The stage of pre-survey

We initially conducted a pre-survey and randomly selected 50 samples, and the following are the descriptive results of the pre-survey. The results of the pre-survey showed that the total score range of the scale is 16–64 (SD: 45.62 ± 11.10). The time to filled in the questionnaire is 3–6 min, with an average of 3.86 min. Supplementary material 3 shows descriptive results of the pre-survey on 50 nursing students.

### Data collection

This study was completed between March and June 2023. The questionnaire included the Chinese version of AGOQ and socio-demographic information. This study adopted multi-stage sampling design. First, Jinzhou Medical University was randomly selected from 6 nursing colleges in Liaoning Province. Next, 50% of classes in each grade (ranging from one to three grade) were selected from the university [21], including the high school undergraduate and vocational college undergraduate students. As a result, 24 classes were selected by the university. In a final step, 25–30 students in each class were selected by cluster sampling. Our investigation was conducted twice, the first was a pre-survey and the second was a formal survey. We distributed questionnaires and collected them on the spot. Everyone can only fill in one questionnaire, and each questionnaire took 3.86 min. Finally, among 696 people, we collected 654 valid questionnaires. Thanks again!

### Statistical analysis

SPSS 25.0, AMOS 23.0, and R 4.3.0 were employed to analyze the statistics. Cronbach’s alpha (α) [22–24] was used to study the internal consistency of the questionnaire and its dimensions.

Exploratory factor analysis (the main component of Varimax rotation) [25, 26] was used to study the structural validity, and its viability was confirmed by Kaiser-Meyer-Olkin test (KMO) and Bartlett test [27, 28]. With EFA, the criteria for the load value of each item is not less than 0.40 on the common factor [29], and the additive contributing rate of the extracted common factors is higher than 40% [30].

To measure model fit in CFA, eight indices were used: chi-square/degree of freedom (χ2/df), goodness of fit index (GFI), adjusted goodness of fit index (AGFI), incremental fit index (IFI), Tucker-Lewis index (TLI) [31], and comparative fit index (CFI). GFI, AGFI, IFI, TLI, and CFI should all be greater than 0.90 [32, 33], and χ2/df should be less than 5 [34].

In order to evaluate the AGOQ, IRT models were used. Graded Response Model (GRM) and Generalized Partial Credit Model (GPCM) [35] were examined for improved model fit using Akek’s information criterion (AIC) and Bayesian information criterion (BIC), whose values are lower suggesting a better model fit [36, 37]. The AIC and BIC values for GPCM in the current study were 27,259 and 27,617, whereas those for GRM were 27,145 and 27,504, respectively. The GRM was used as a result since it had a better model fit. For each item, the discrimination parameters (α) and difficulty parameters (β) were estimated. Additionally, item characteristic curves, item information curves, and total (scale) information Curves were measured [38, 39]. The larger the area covered under the curves; the item can more accurately estimate nursing students’ academic goal orientations.

## Results

### Descriptive statistics

Table 1 shows the descriptive results of the questionnaire. Of the participating 654 nursing students, the ages ranged from 17 to 26 years, with an average of (21.61 ± 1.73). Most of them were females (568,86.85%), sophomores (430, 65.75%), and living in urban (342, 52.29%).

Table 2 shows descriptive results of the AGOQ by sex and grade. In the questionnaire, the average score for learning or task goal is the highest (mean = 3.59, SD = 1.05), and the average score for ego self- frustration goal is the lowest (mean = 2.60, SD = 1.08).

**Table 1:** Frequency distribution of demographic characteristics(n = 654)

Variables	Groups	N	$\% /\bar X \pm S$
City	Urban	342	52.29
	Rural	253	38.69
	Suburbs	59	9.02
Sex	Male	86	13.15
	Female	568	86.85
Age (years)	17–26		21.61 ± 1.73
Grade	Freshman	297	45.41
	Sophomore	267	40.83
	Junior	90	13.76

**Table 2:** Descriptive results of the Academic Goals Orientation Questionnaire by sex and grade

Dimensions and items	Sex				Grade							*P*
	Male		Female		*P*	Freshman		Sophomore		Junior		
	mean	sd	mean	sd		mean	sd	mean	sd	mean	sd	
F1	2.60	1.08	2.57	0.95	0.031	2.83	1.05	2.50	0.96	2.68	0.92	0.010
Item4	2.59	1.14	2.67	1.07	0.393	2.93	1.20	2.58	1.07	2.76	1.03	0.020
Item7	2.55	1.16	2.69	1.09	0.158	2.88	1.11	2.59	1.10	2.82	1.07	0.020
Item11	2.63	1.19	2.51	1.08	0.071	2.79	1.10	2.45	1.09	2.61	1.08	0.030
Item 14	2.64	1.25	2.42	1.03	0.001	2.71	1.19	2.38	1.05	2.54	1.02	0.030
F2	3.00	0.92	3.08	0.82	0.716	3.07	0.84	3.10	0.84	2.98	0.80	0.291
Item 2	3.03	1.20	3.15	0.97	0.013	3.16	0.99	3.17	1.03	3.03	0.96	0.292
Item 6	2.98	1.18	3.16	0.98	0.118	3.16	1.03	3.19	1.00	2.98	1.02	0.079
Item 10	3.08	1.11	3.02	1.02	0.388	3.07	1.04	3.04	1.03	2.99	1.01	0.845
Item 13	2.90	1.12	2.99	1.00	0.165	2.87	0.98	3.02	1.03	2.93	0.99	0.411
F3	2.97	0.96	2.82	0.78	0.091	2.97	0.90	2.78	0.80	2.94	0.77	0.047
Item 3	3.08	1.25	2.87	1.10	0.127	3.07	1.14	2.85	1.13	2.95	1.10	0.247
Item 8	2.94	1.14	2.85	1.06	0.953	2.96	1.01	2.80	1.10	3.00	1.00	0.097
Item 12	2.94	1.22	2.78	1.01	0.029	2.94	1.16	2.74	1.02	2.90	1.04	0.131
Item 15	2.92	1.22	2.79	1.02	0.120	2.90	1.12	2.75	1.04	2.91	1.04	0.200
F4	3.59	1.05	3.72	0.84	0.003	3.49	0.92	3.75	0.84	3.68	0.90	0.062
Item 1	3.60	1.10	3.74	0.93	0.012	3.57	0.98	3.75	0.92	3.70	1.01	0.330
Item 5	3.60	1.16	3.71	0.93	0.001	3.40	1.02	3.75	0.93	3.70	1.01	0.021
Item 9	3.60	1.19	3.76	0.91	0.000	3.49	1.06	3.77	0.91	3.76	1.00	0.069
Item 16	3.55	1.16	3.68	0.97	0.011	3.49	1.04	3.73	0.99	3.58	0.98	0.087

F1(Self- frustration goal, items 4, 7, 11, 14), F2(Ego self- enhancement goal, items 2, 6, 10, 13), F3(Work avoidance goal, items 3, 8, 12, 15), and F4(Learning or task goals, items 1, 5, 9, 16)

### Reliability

Table 3 shows Cronbach’s coefficient alpha for each item. According to the results of the reliability analysis, it can be seen that the standardized reliability coefficient of the Chinese version of AGOQ is 0.859, and the questionnaire is generally reliable. Cronbach’s Alpha value of each item after deleting the item is less than 0.859 of the whole, so no adjustment is needed.

**Table 3:** Cronbach’s coefficient alpha(n = 654, α = 0.05)

Items	Drop if	r dropped	r
Item4	0.850	0.503	0.586
Item7	0.849	0.534	0.615
Item11	0.852	0.470	0.558
Item14	0.851	0.486	0.571
Item2	0.847	0.568	0.638
Item6	0.850	0.514	0.591
Item10	0.847	0.562	0.634
Item13	0.850	0.517	0.594
Item3	0.854	0.435	0.530
Item8	0.854	0.420	0.511
Item12	0.855	0.412	0.502
Item15	0.854	0.429	0.519
Item1	0.851	0.498	0.573
Item5	0.851	0.491	0.567
Item9	0.851	0.485	0.560
Item16	0.853	0.454	0.536

Drop if: Cronbach alpha when the item is removed; r dropped: item-total correlation without the item; r: item-total (point-biserial) correlation

### Validity

#### Construct validity

##### Exploratory factor analysis

**Fig. 1:**
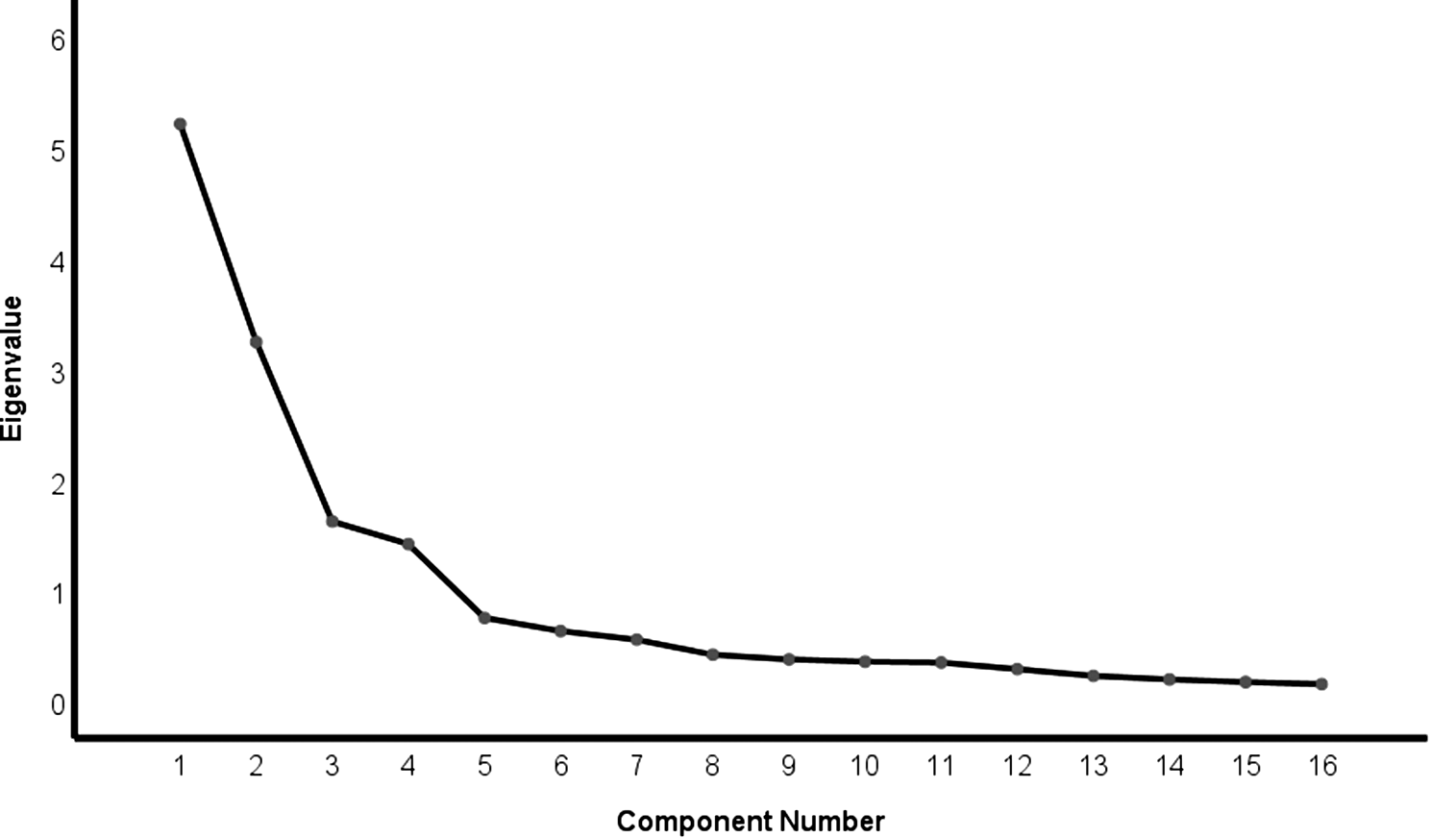
Scree plot

Table 4 shows the rotation sums of squared loadings. The Kaiser-Meyer-Olkin (KMO) test was 0.848, and Bartlett sphericity test was significant (*χ*^*2*^ = 6157.990; *P* < 0.001) [30]. The exploratory factor analysis (EFA) analysis, revealed four dimensions through the scree plot and eigenvalue (> 1.0) [40]. Four factors supported by the scree plot (Fig. 1) accounted for 71.892% of the variance, respectively explaining 20.256%, 19.788%, 17.099% and 14. 748%.

Table 5 shows factor load and communalities of each item in AGOQ of 16 items. According to the type of academic goal orientation, the items are divided into four dimensions, and the dimensions of the Chinese version of AGOQ are the same as those of the original version, namely (i) Ego self-frustration goal (items 4, 7, 11, 14); (ii) Ego self-enhancement goal (items 2, 6, 10, 3); (iii) Work avoidance goal (items 3, 8, 12, 15); and (iv) learning or task goals (items 1, 5, 9, 16). As a result, the load value of each project on one of the common factors is higher than 0.40, and there is no double load phenomenon [41].

**Table 4:** Rotation Sums of Squared Loadings

Model	of Variance (%)				
	Ego self- frustration goal	Ego self- enhancement goal	Work avoidance goal	Learning goal dimension	the Total Variance
Initial model	17.182	14.486	11.148	10.682	53.498
Modified model	20.256	19.788	17.099	14.748	71.892

Kaiser-Meyer-Olkin Measure of Sampling Adequacy = 0.848, Bartlett’s Test of Sphericity, Approx. Chi-Square = 6157.990, *P* < 0.001

**Table 5:** Factor load and communalities of each item in AGOQ of 16 Items(n = 654)

Items	F1	F2	F3	F4	Communalities
Item 7	**0.898**	0.016	0.200	0.043	0.848
Item 4	**0.887**	-0.006	0.216	0.087	0.841
Item 11	**0.859**	0.016	0.276	0.012	0.814
Item 14	**0.843**	-0.029	0.118	0.178	0.757
Item 6	0.066	**0.899**	0.088	0.157	0.845
Item 13	-0.059	**0.880**	0.126	0.135	0.813
Item 2	0.055	**0.858**	0.076	0.164	0.772
Item 10	-0.067	**0.834**	0.166	0.175	0.758
Item 9	0.230	0.023	**0.835**	0.063	0.755
Item 5	0.185	0.163	**0.790**	0.011	0.685
Item 16	0.186	0.099	**0.783**	0.193	0.695
Item 1	0.176	0.178	**0.702**	0.200	0.596
Item 15	0.011	0.165	0.045	**0.842**	0.739
Item 12	0.058	0.147	0.033	**0.791**	0.652
Item 8	0.068	0.130	0.120	**0.721**	0.555
Item 3	0.158	0.124	0.239	**0.531**	0.380

F1(Self- frustration goal, items 4, 7, 11, 14), F2(Ego self- enhancement goal, items 2, 6, 10, 13), F3(Work avoidance goal, items 3, 8, 12, 15), and F4(Learning or task goals, items 1, 5, 9, 16)

##### Confirmatory factor analysis

**Fig. 2:**
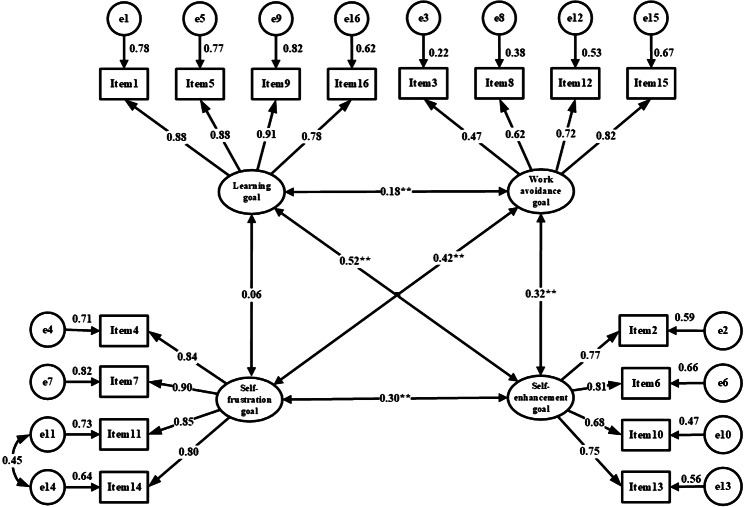
Standardized four-factor structural model of the Chinese version of the Academic goals orientation questionnaire (n = 654); F1(Self- frustration goal, items 4, 7, 11, 14), F2(Ego self- enhancement goal, items 2, 6, 10, 13), F3(Work avoidance goal, items 3, 8, 12, 15), and F4(Learning or task goals, items 1, 5, 9, 16)

**Fig. 3:**
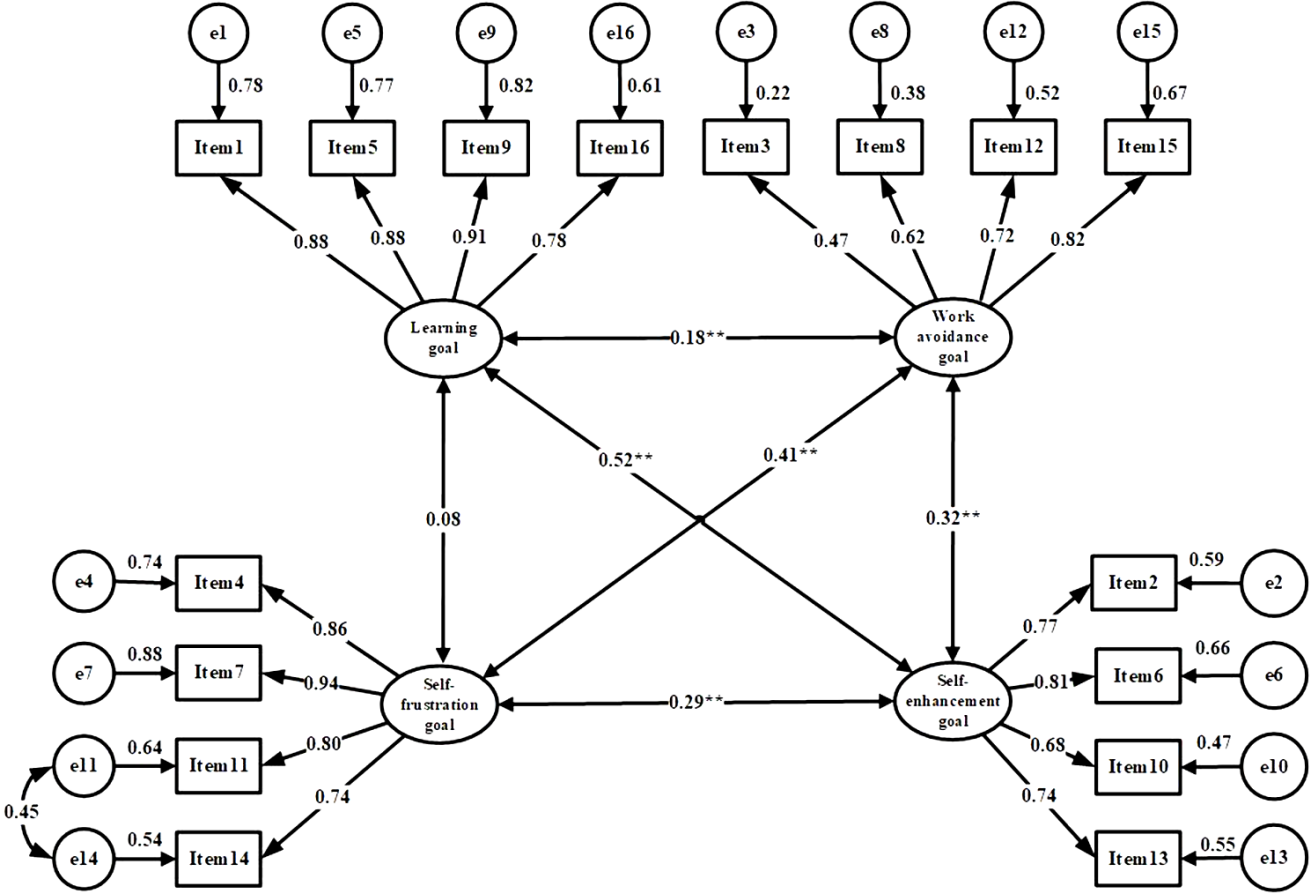
Standardized four-factors structural model of the modified Chinese version of the Academic goals orientation questionnaire (n = 654); F1(Self- frustration goal, items 4, 7, 11, 14), F2(Ego self- enhancement goal, items 2, 6, 10, 13), F3(Work avoidance goal, items 3, 8, 12, 15), and F4(Learning goal dimension, items 1, 5, 9, 16)

The results of confirmatory factor analysis (CFA) are shown in Table 6. With CFA, in an original four-factor model with the Chinese version of the AGOQ, the fit indices were not acceptable (Table 6 and Fig. 2). Then, modification indices were taken to improve the fit indices, and a new four-factors model was built and showed an acceptable goodness-of-fit [34, 42–45], chi-square/degree of freedom(CMIN/DF) = 4.008, goodness of fit index (GFI) = 0.932, adjusted goodness of fit index (AGFI) = 0.905, comparative fit index (CFI) = 0.952, incremental fit index(IFI) = 0.952, Tucker Lewis index (TLI) = 0.941. (Table 6 and Fig. 3).

**Table 6:** Evaluation of fitness of SEM model

Model	CMIN/DF	NFI	RFI	IFI	TLI	CFI	RMR	GFI	AGFI	PGFI	PRATIO	PNFI	PCFI
Initial model	5.010	0.921	0.903	0.936	0.921	0.936	0.061	0.912	0.878	0.657	0.817	0.752	0.764
Modified model	4.008	0.937	0.923	0.952	0.941	0.952	0.062	0.932	0.905	0.665	0.808	0.758	0.770
Standard value	< 5.000	> 0.900	> 0.900	> 0.900	> 0.900	> 0.900	> 0.500	> 0.500	> 0.500	> 0.500	> 0.500	> 0.500	> 0.500

### Discriminant validity

In our study, the scores of the top (50%) and low (50%) groups were analyzed using a two-tailed independent samples t-test. As can be seen in Table 7, the difference in all scores between the two groups reached the significant level (*P* < 0.001).

**Table 7:** Discriminant validity analysis in AGOQ (n = 654)

Item	Low-score group mean ± *SD*	High-score group mean ± *SD*	*t*	*P*
F1	1.68 ± 0.52	3.47 ± 0.63	-39.708	< 0.001
Item4	1.76 ± 0.61	3.55 ± 0.60	-37.910	< 0.001
Item7	1.77 ± 0.62	3.58 ± 0.62	-37.452	< 0.001
Item11	1.61 ± 0.49	3.44 ± 0.68	-39.671	< 0.001
Item 14	1.59 ± 0.49	3.31 ± 0.74	-34.875	< 0.001
F2	2.32 ± 0.69	3.82 ± 0.62	-29.411	< 0.001
Item 2	2.37 ± 0.70	3.90 ± 0.61	-29.662	< 0.001
Item 6	2.38 ± 0.70	3.90 ± 0.65	-28.743	< 0.001
Item 10	2.28 ± 0.73	3.79 ± 0.66	-27.649	< 0.001
Item 13	2.24 ± 0.70	3.72 ± 0.68	-27.301	< 0.001
F3	2.02 ± 0.67	3.66 ± 0.67	-31.620	< 0.001
Item 3	2.02 ± 0.68	3.77 ± 0.72	-31.981	< 0.001
Item 8	2.06 ± 0.72	3.67 ± 0.69	-29.063	< 0.001
Item 12	1.99 ± 0.64	3.61 ± 0.67	-31.747	< 0.001
Item 15	2.00 ± 0.69	3.61 ± 0.67	-30.151	< 0.001
F4	3.03 ± 0.83	4.38 ± 0.48	-25.579	< 0.001
Item 1	3.07 ± 0.85	4.38 ± 0.49	-24.223	< 0.001
Item 5	3.01 ± 0.83	4.39 ± 0.49	-25.909	< 0.001
Item 9	3.08 ± 0.83	4.40 ± 0.49	-24.763	< 0.001
Item 16	2.96 ± 0.86	4.37 ± 0.48	-25.829	< 0.001

F1(Self- frustration goal, items 4, 7, 11, 14), F2(Ego self- enhancement goal, items 2, 6, 10, 13), F3(Work avoidance goal, items 3, 8, 12, 15), and F4(Learning or task goals, items 1, 5, 9, 16)

### Item response theory models

In order to evaluate the AGOQ, IRT models were used. Graded Response Model (GRM) and Generalized Partial Credit Model (GPCM) were examined for improved model fit using AIC and BIC, whose values are lower suggesting a better model fit. The AIC and BIC values for GPCM in the current study were 27,259 and 27,617, whereas those for GRM were 27,145 and 27,504, respectively. The GRM was used as a result since it had a better model fit. According to Table 8, the range of all item discrimination factors was between 0.237 and 3.689. The parameters for difficulty ranged from − 16.603 to 6.460.

**Table 8:** Estimates of discrimination and threshold parameters for the Scale under the graded response model with the Graded Response Model(n = 654, α = 0.05)

Items	Threshold				Discrimination
	β_1_	β_2_	β_3_	β_4_	α_i_
Item4	-1.073	0.034	0.868	2.290	3.300
Item7	-0.998	0.008	0.816	2.110	3.689
Item11	-0.891	0.167	0.951	2.230	3.267
Item14	-0.857	0.285	1.030	2.430	3.157
Item2	-3.447	-1.224	0.830	3.300	0.913
Item6	-4.349	-1.716	1.076	3.910	0.691
Item10	-2.739	-0.993	0.997	3.080	0.991
Item13	-3.258	-1.043	1.268	3.560	0.867
Item3	-2.870	-0.539	1.347	3.440	0.780
Item8	-2.651	-0.667	1.542	3.760	0.823
Item12	-2.688	-0.387	1.534	3.750	0.881
Item15	-2.386	-0.377	1.492	3.560	0.948
Item1	-14.584	-9.753	-2.692	6.460	0.237
Item5	-14.214	-8.639	-2.109	6.240	0.253
Item9	-16.603	-9.918	-2.790	6.260	0.241
Item16	-14.155	-6.674	-1.432	5.450	0.294

**Fig. 4:**
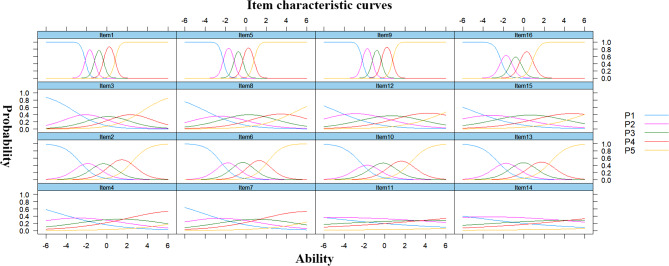
Item characteristic curves

**Fig. 5:**
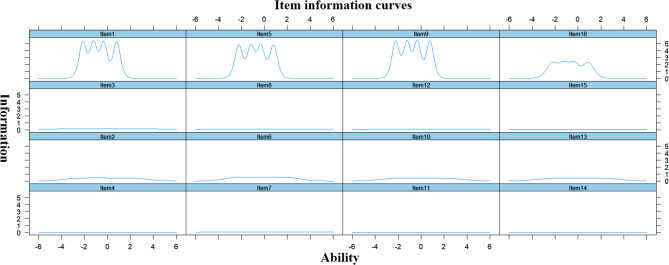
Item information curves

**Fig. 6:**
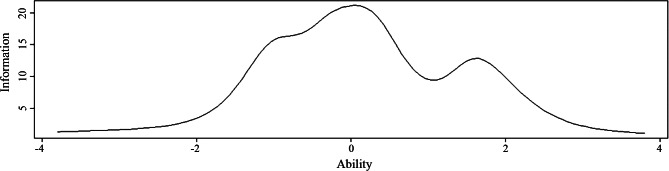
Total (scale) information curve

The item characteristic curves and item information curves for the Chinese AGOQ are shown in Figs. 4 and 5, respectively. The curves of the Item characteristic curves showed that the order of categories’ thresholds for all the items was as expected, which meant that all categories were adequate in terms of placing a respondent on the scale. The distributions of the item information curves were multimodal. The shapes of items 1, 5, 9 and 16 were the steepest and provided more information than the other items. Figure 6 is the total scale information curve. The peak value of the curve is between − 1 and 1, which means that nursing students with ability level between − 1 and 1 get the most information through AGOQ scale evaluation. This shows that AGOQ scale has the strongest ability to distinguish the academic goal orientation of nursing students with abilities.

## Discussion

The literature in nursing research links personal characteristics (such as child care or cultural differences) and other factors (such as study intensity, clinical practice, or a lack of a consulting plan) with academic burnout [46], dropping out of school, or achieving and maintaining academic goals [47, 48]. However, little research has been conducted on education, particularly on the sorts of academic aim orientation of nursing students in China.

As far as we know, this is the first study on academic goals orientation questionnaire (AGOQ) among nursing students in China based on structural equation modeling (SEM) and item response theory (IRT) model. The test results show that the Chinese version of AGOQ has good psychometric characteristics and is an effective and reliable tool. These results are consistent with the original version developed by Skaalvik [4] and the academic goals orientation questionnaire verification conducted by Navea Martin [8] in Spain.

Previously, Elliot [49] developed and verified a similar questionnaire among psychology students. March [50] used this questionnaire consisting of the same dimensions with three items per dimension among US nursing students, but the authors did not report its psychometric properties in the sample studied. Some scholars [14, 51] used other language versions of the questionnaire, and also obtained sufficient internal consistency among nursing students (α = 0.82 and α = 0.85). Although the questionnaire showed good internal consistency, it did not examine the psychometric properties. Therefore, the present study decided to use the questionnaire developed and verified by Skaalvik [4], because the Spanish version of psychometrics has been verified by scholars before [8].

In the exploratory factor analysis (EFA) model, this study extracted four factors which are the same as the original scale. The four factors explained 71.892% of the total variance, 20.256%, 19.788%, 17.099% and 14.748%, respectively. The measured values of the model fit well (chi-square/degree of freedom (CMIN/DF) = 4.008, comparative fit index (CFI) = 0.952, incremental fit index (IFI) = 0.952, Tucker Lewis index (TLI) = 0.941). The results showed that the model has strong factor load and explanatory difference. The results of confirmatory factor analysis (CFA) confirmed that the Chinese version of AGOQ had a fitting index. There was significant difference in discriminant validity between the high group and the low group (*P* < 0.001). In addition, each item of AGOQ has higher load value and commonality coefficient. The results also indicated that there were strong factor loadings and explained variance in the structural equation modeling, consistent with the EFA results.

Significant differences are rarely found in the analysis of the dimensions and items of the questionnaire. The score of learning and task goal dimension is the only dimension with significant gender difference. This is consistent with previous scholars’ research [51], that is, women scored significantly higher in learning or task goals. With regard to work avoidance, freshmen scored significantly higher in job avoidance dimension than other grades. Students pursuing a work avoidance objective have been defined as individuals who constantly avoid putting in effort to meet exceptional levels of achievement, doing only the bare minimum to get by, and avoiding difficult activities [12, 52]. When freshmen enter a new learning environment, they may avoid trying difficult jobs because of their low adaptability. Among college students in China, there is a very interesting phenomenon “Buddhist-Style college students” [53], who had hoped that they could relax in college and not worry too much.

In addition, through IRT analysis, AGOQ has certain discriminating ability, and all discriminating parameters are higher than 0.2, indicating that AGOQ is easy to distinguish the academic goal orientation of nursing students in China. In terms of difficulty, the difficulty is increasing monotonically, which indicates that AGOQ has acceptable difficulty. In total scale information curve, the peak value of the curve is between − 1 and 1, which means that nursing students with ability level between − 1 and 1 get the most information through AGOQ evaluation. This shows that AGOQ has the strongest ability to distinguish the academic goal orientation of nursing students with abilities around − 1 to 1.

### Limitations

Some restrictions should also be considered. Firstly, a cross-sectional study was carried out in our study, so further longitudinal study is needed to confirm these results. Secondly, The sample of this study comes from a nursing school in Liaoning Province, China. The results of this study have regional limitations, so they can’t be generalized among nursing students in China. Therefore, further efforts should be made to expand the sample coverage and take into account the adaptability of different groups and hope to continue to verify the feasibility of the subscale in other areas of China in future research. Despite these limitations, the current research can be considered as groundbreaking research. Specifically, this study is the first time that China has used SEM and IRT models to measure the psychometric characteristics of AGOQ.

## Conclusions

This study tested the psychometric characteristics of AGOQ of nursing students in China. The results confirmed that China version of AGOQ has good psychometric characteristics and can be used to measure the academic goal orientation of nursing students in China.

### Electronic supplementary material

Below is the link to the electronic supplementary material.

**Supplementary Material 1:** The training guidance of investigators

**Supplementary Material 2:** The Academic Goals Orientation Questionnaire

**Supplementary Material 3:** Descriptive results of the pre-survey on 50 nursing student (N=50)

## Data Availability

The datasets generated and/or analyzed during the present study are not publicly available to preserve the anonymity of the participants but are available from the corresponding author at reasonable request.

## Abbreviations

AGFI: Adjusted goodness of fit index
AGOQ: Academic goals orientation questionnaire
AIC: Akek’s information criterion
BIC: Bayesian information criterion
CFA: Confirmatory factor analysis
CFI: Comparative fit index
CMIN/DF: Chi-square/degree of freedom
EFA: Exploratory factor analysis
GFI: Goodness of fit index
GPCM: Generalized Partial Credit Model
GRM: Graded Response Model
IFI: Incremental fit index
IRT: Item response theory
KMO: Kaiser-Meyer-Olkin
SEM: Structural equation modeling
TLI: Tucker Lewis index
