# Supplementary material for: Validation of the Chinese version of academic goals orientation questionnaire in nursing student: a study based on SEM and IRT multidimensional models
Source: BMC Nurs. 2023 Dec 6;22:465. doi: 10.1186/s12912-023-01630-0 (PMC10698999; doi:10.1186/s12912-023-01630-0)
Supplement: Supplementary file 1 — Supplementary Material 1: The training guidance of investigators [file 12912_2023_1630_MOESM1_ESM.docx]

Supplementary material 1 The training guidance of investigators

| **Training guidance of investigators** | |
| --- | --- |
| **Investigator** | 10 full-time graduate students with nursing education background |
| **Investigative steps** | **Step 1:** Before distributing the questionnaire, discuss the conversation skills. |
|  | **Step 2:** Obtain the informed consent of participants and sign the informed consent form. |
|  | **Step 3:** Explain the matters needing attention in filling out the questionnaire, and explain it to the participants in an easy-to-understand language. |
|  | **Step 4:** Ensure the on-site distribution and collection of questionnaires. |
|  | **Step 5:** Pre-survey-distribute 30 questionnaires first, check the reliability and validity after recycling, and adjust them in time when problems are found. |
|  | **Step 6:** Formal investigation |
| **Quality inspection** | **1.** Reject unqualified questionnaires with missing items and regular and continuous options. |
|  | **2.** The two people respectively enter the data in the same order, and then review and compare them (validity test), and then verify and modify the inconsistent data entered by the two people against the original questionnaire until the data in the two database files are completely consistent, so as to avoid the input errors caused by human factors in the data entry process. |
