# Supplementary material for: Validation of the Chinese version of academic goals orientation questionnaire in nursing student: a study based on SEM and IRT multidimensional models
Source: BMC Nurs. 2023 Dec 6;22:465. doi: 10.1186/s12912-023-01630-0 (PMC10698999; doi:10.1186/s12912-023-01630-0)
Supplement: Supplementary file 2 — Supplementary Material 2: The Academic Goals Orientation Questionnaire [file 12912_2023_1630_MOESM2_ESM.docx]

**Supplementary material 2 The Academic Goals Orientation Questionnaire**

| **Items** | **Item content** | **Score** |
| --- | --- | --- |
|  | **Self ego self-frustration goal** |  |
| **Item4** | 4. When I answer questions that are asked in class, I am worried about what my classmates are going to think. | 1 2 3 4 5 |
| **Item7** | 7. When I participate in an activity, I am worried about what my classmates may be thinking about me. | 1 2 3 4 5 |
| **Item11** | 11. In class, I worry about being ridiculed | 1 2 3 4 5 |
| **Item 14** | 14. When I answer incorrectly in class, what worries me the most is what my classmates think of me. | 1 2 3 4 5 |
|  | **Self ego self-enhancement goal** |  |
| **Item 2** | 2. Succeeding in these studies is doing homework better than other students | 1 2 3 4 5 |
| **Item 6** | 6. I try to get higher grades than other students. | 1 2 3 4 5 |
| **Item 10** | 10. It is important for me to know how to do tasks that other classmates do not know. | 1 2 3 4 5 |
| **Item 13** | 13. In studies, I always try to do better than other students. | 1 2 3 4 5 |
|  | **Work avoidance goal** |  |
| **Item 3** | 3. I do not want work to be done at home. | 1 2 3 4 5 |
| **Item 8** | 8. I prefer the subjects in which I do not have to work. | 1 2 3 4 5 |
| **Item 12** | 12. In class, I prefer to do as little as possible. | 1 2 3 4 5 |
| **Item 15** | 15.I try to avoid difficult tasks or subjects | 1 2 3 4 5 |
|  | **Learning or task goal** |  |
| **Item 1** | 1. It is important for me to learn new things | 1 2 3 4 5 |
| **Item 5** | 5.I am concerned about improving my skills/abilities in class. | 1 2 3 4 5 |
| **Item 9** | 9.It is important for me to learn to solve the problems that are proposed. | 1 2 3 4 5 |
| **Item 16** | 16.In class, I like to learn interesting things. | 1 2 3 4 5 |
