# Supplementary material for: Validation of the Chinese version of academic goals orientation questionnaire in nursing student: a study based on SEM and IRT multidimensional models
Source: BMC Nurs. 2023 Dec 6;22:465. doi: 10.1186/s12912-023-01630-0 (PMC10698999; doi:10.1186/s12912-023-01630-0)
Supplement: Supplementary file 3 — Supplementary Material 3: Descriptive results of the pre-survey on 50 nursing student (N=50) [file 12912_2023_1630_MOESM3_ESM.docx]

**Supplementary material 3 Descriptive results of the pre-survey on 50 nursing student (N=50)**

| **Category** | **Score** | | ***Mean±SD*** |
| --- | --- | --- | --- |
|  | **Min** | **Max** |  |
| Item 4 | 1 | 5 | 2.52±1.11 |
| Item 7 | 1 | 5 | 2.48±1.16 |
| Item 11 | 1 | 5 | 2.40±1.14 |
| Item 14 | 1 | 4 | 2.20±1.05 |
| Item 2 | 1 | 5 | 2.98±1.15 |
| Item 6 | 1 | 5 | 3.10±1.13 |
| Item 10 | 1 | 5 | 2.98±1.10 |
| Item 13 | 1 | 5 | 2.94±1.20 |
| Item 3 | 1 | 4 | 2.40±0.93 |
| Item 8 | 1 | 5 | 2.34±0.96 |
| Item 12 | 1 | 5 | 2.56±0.99 |
| Item 15 | 1 | 5 | 2.56±0.97 |
| Item 1 | 1 | 5 | 3.48±1.09 |
| Item 5 | 1 | 5 | 3.60±1.12 |
| Item 9 | 1 | 5 | 3.66±1.08 |
| Item 16 | 1 | 5 | 3.42±1.01 |
| Total score | 16 | 64 | 45.62±11.10 |
| Spent time (minute) | 3 | 6 | 3.86±0.96 |
| F1(Self- frustration goal, items 4, 7, 11, 14), F2(Ego self- enhancement goal, items 2, 6, 10, 13), F3(Work avoidance goal, items 3, 8, 12, 15), and F4(Learning or task goals, items 1, 5, 9, 16). | | | |
